# Relationship between stroke etiology and collateral status in anterior circulation large vessel occlusion

**DOI:** 10.1007/s00415-020-10009-z

**Published:** 2020-06-25

**Authors:** Eva Hassler, Markus Kneihsl, Hannes Deutschmann, Nicole Hinteregger, Marton Magyar, Ulrike Wießpeiner, Melanie Haidegger, Simon Fandler-Höfler, Sebastian Eppinger, Kurt Niederkorn, Christian Enzinger, Franz Fazekas, Thomas Gattringer

**Affiliations:** 1 Division of Neuroradiology, Vascular and Interventional Radiology, Department of Radiology, Medical University of Graz, Graz, Austria; 2 Department of Neurology, Medical University of Graz, Auenbruggerplatz 22, 8036 Graz, Austria

**Keywords:** Stroke, Collateral circulation, Thrombectomy, Carotid artery diseases, Outcome

## Abstract

**Background and purpose:**

Clinical outcome after mechanical thrombectomy (MT) for large vessel occlusion (LVO) stroke is influenced by the intracerebral collateral status. We tested the hypothesis that patients with preexisting ipsilateral extracranial carotid artery stenosis (CAS) would have a better collateral status compared to non-CAS patients. Additionally, we evaluated MT-related adverse events and outcome for both groups.

**Methods:**

Over a 7-year period, we identified all consecutive anterior circulation MT patients (excluding extracranial carotid artery occlusion and dissection). Patients were grouped into those with CAS ≥ 50% according to the NASCET criteria and those without significant carotid stenosis (non-CAS). Collateral status was rated on pre-treatment CT- or MR-angiography according to the Tan Score. Furthermore, we assessed postinterventional infarct size, adverse events and functional outcome at 90 days.

**Results:**

We studied 281 LVO stroke patients, comprising 46 (16.4%) with underlying CAS ≥ 50%. Compared to non-CAS stroke patients (*n* = 235), patients with CAS-related stroke more often had favorable collaterals (76.1% vs. 46.0%). Recanalization rates were comparable between both groups. LVO stroke patients with underlying CAS more frequently had adverse events after MT (19.6% vs. 6.4%). Preexisting CAS was an independent predictor for favorable collateral status in multivariable models (Odds ratio: 3.3, *p* = 0.002), but post-interventional infarct size and functional 90-day outcome were not different between CAS and non-CAS patients.

**Conclusions:**

Preexisting CAS ≥ 50% was associated with better collateral status in LVO stroke patients. However, functional 90-day outcome was independent from CAS, which could be related to a higher rate of adverse events.

## Introduction

Mechanical thrombectomy (MT) is the recommended treatment for acute ischemic stroke due to large vessel occlusion (LVO) of the anterior cerebral circulation [1]. With increasing experience and technical advances, successful recanalization can nowadays be achieved in up to 90% of all thrombectomy cases. However, successful recanalization does not always entail a favorable outcome after endovascular stroke treatment. In this context, the extent of leptomeningeal collateral perfusion has been identified as a major determinant of patients’ clinical prognosis [2]. Unfavorable collateral status on preinterventional angiography has been related to larger final infarct volumes and consequently a worse clinical outcome after MT [2, 3].

Previously, it has been assumed that chronically developing extracranial carotid artery stenosis (CAS) could enhance cerebral collateral flow, which might be related to a more favorable prognosis in acute stroke patients [4]. This raised the question, whether an underlying CAS would also lead to better leptomeningeal collaterals in patients with acute anterior circulation LVO stroke compared to those with more abrupt vessel occlusion due to proximal embolism (e.g. cardiogenic embolism from atrial fibrillation).

While small previous investigations showed inconsistent results on the predictive value of stroke etiology on collateral status in LVO patients, [5–7] a very recent subanalysis of the MR-CLEAN Registry reported higher collateral recruitment in acute stroke patients with an underlying atherosclerotic carotid artery stenosis [8]. However, for yet unknown reasons, this might not translate into a higher chance for CAS patients to achieve functional independency after stroke [8].

We aimed at investigating preinterventional leptomeningeal collateral status in acute LVO patients according to the underlying putative stroke mechanism (atherosclerotic CAS ≥ 50% versus patients without significant ipsilateral CAS) and how this affects postinterventional adverse events and clinical outcome.

## Materials and methods

### Patient selection and data collection

For the present study, we identified all consecutive ischemic stroke patients aged ≥ 18 years, who were treated by MT for acute anterior circulation LVO (i.e. occlusion of the intracranial internal carotid artery or middle cerebral artery in the M1 or M2 segment) between 2010 and 2017 at our primary and tertiary care university hospital.

Clinical data including demographics, cerebrovascular risk factors, stroke etiology, characteristics of the endovascular procedure and outcome were retrieved from our prospectively collected electronical thrombectomy database [9].

**Fig. 1:**
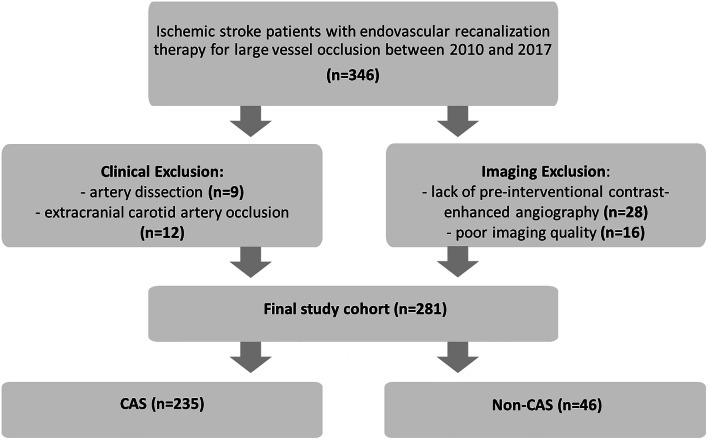
Flow diagram of included study participants

Patients were divided into those with an underlying atherosclerotic extracranial ipsilateral carotid artery stenosis ≥ 50% (CAS) and those without an indication of preexisting significant carotid steno-occlusive disease (non-CAS). The cut-off was chosen because it (1) corresponds to recent guideline recommendations for diagnosing symptomatic carotid artery stenosis [10] and (2) was also used in prior studies on this topic [8]. Presence and degree of stenosis was determined on preinterventional computed tomography (CT) or magnetic resonance imaging (MRI) based contrast enhanced (CE) angiography and confirmed by digital subtraction angiography during the thrombectomy procedure using the North American Symptomatic Carotid Endarterectomy Trial (NASCET) criteria [11]. Patients with extracranial carotid artery occlusion were excluded from the study, as it was not possible to determine whether they had a preexisting stenosis (Fig. 1).

Mechanical thrombectomy was conducted by interventional radiologists using stent retrievers and/or aspiration systems. If ipsilateral CAS was present, acute stenting procedures were performed at the discretion of the treating physician depending on morphology and grade of carotid stenosis (e.g. high-risk stenosis due to ulcerated plaque, visible residual thrombi or filiform stenosis).

### Imaging work-up and analyzes

All included patients underwent preinterventional brain imaging including intra- and extracranial CT or MRI based CE angiography (CT angiography: ≈ 90%).

Postinterventional control brain imaging (predominantly MRI) was routinely performed 24 h after thrombectomy or at any time in case of clinical deterioration.

All images were retrospectively analyzed by two experienced neuroradiologists (E.H., M.M.), who were blinded to clinical and outcome data.

Leptomeningeal collateral status on preinterventional CT- or MR-angiography was categorized according to the collateral score by Tan et al. into scores 0: absent collateral supply of the affected MCA territory, 1: collateral supply filling ≤ 50%, 2: collateral supply filling > 50% but < 100%, and 3:100% collateral supply of the occluded MCA territory [12].

**Fig. 2:**
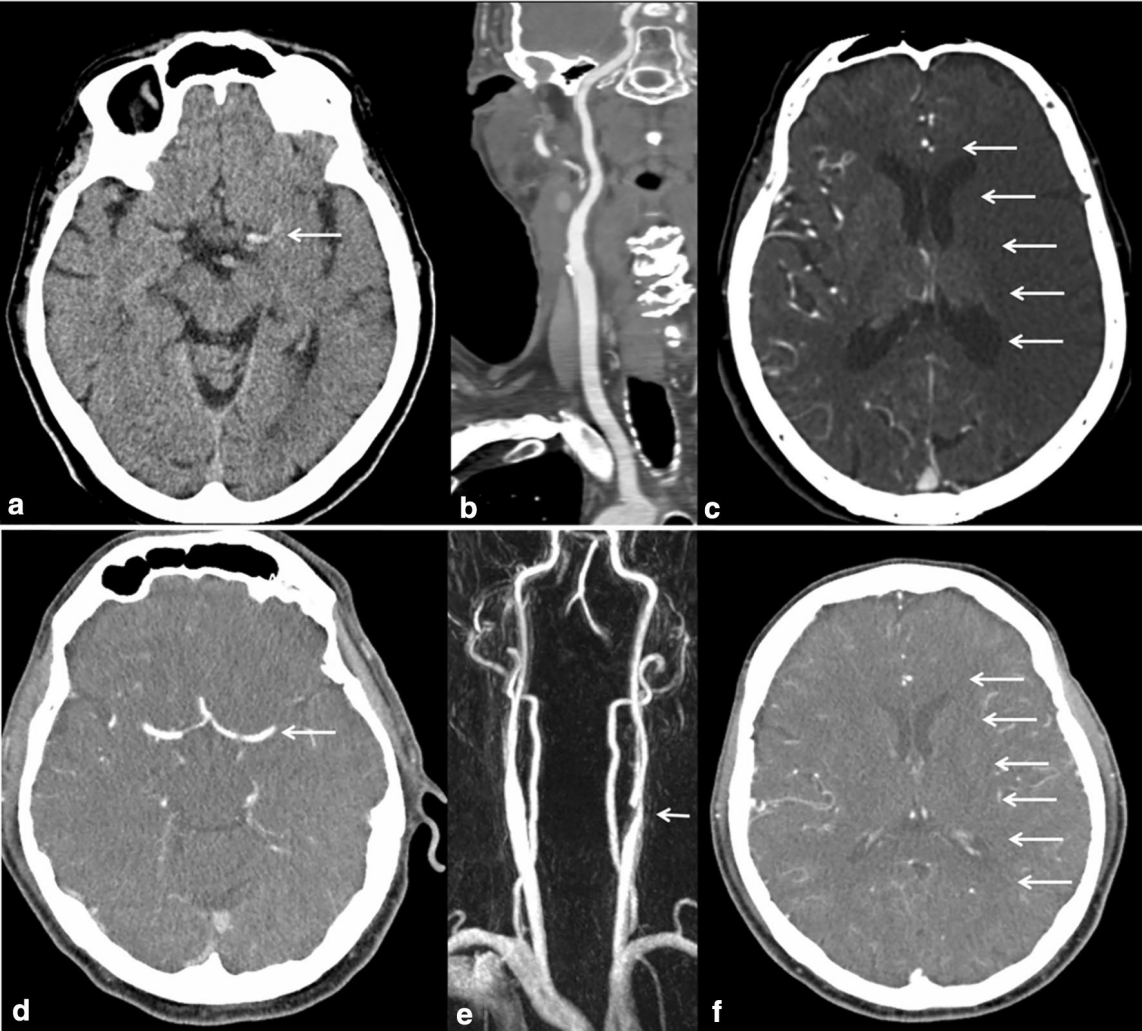
Exemplary cases of acute LVO stroke patients with good and poor collaterals. **a** Shows left sided hyperdense media sign (arrow) on preinterventional CT scan of a patient with acute occlusion of the M1 segment of the middle cerebral artery without an indication of ipsilateral carotid artery stenosis (**b**). Intracranial CT angiography (CTA) displays unfavorable collateral status according to a Tan collateral score of 0 before thrombectomy (**c**, arrows). Another patient presents with acute left sided atheroembolic M1 occlusion (**d**, arrow) and high-grade extracranial carotid artery stenosis on preinterventional CTA, which is demonstrated in **e** (arrow). **f** shows favorable collaterals according to a Tan collateral score of 3 (arrows)

Leptomeningeal collaterals were furthermore dichotomized into unfavorable collaterals (collateral score: 0–1) and favorable collaterals (collateral score: 2–3) (Fig. 2) [5].

### Postinterventional adverse events and outcome

Postinterventional brain scans were reviewed to identify intracranial hemorrhage (ICH). ICH after MT was defined according to the Heidelberg Bleeding Classification and deemed symptomatic if a deterioration of patient’s clinical symptoms was observed (defined as a National Institutes of Health Stroke Scale [NIHSS] score increase of > 2 points in one category or > 4 points in total) [13, 14]. Treatment-related arterial re-occlusion or dissection was diagnosed by digital subtraction angiography in synopsis with postinterventional CT/MR-angiography and color-coded duplex sonography of the extra- and intracranial vessels.

Functional neurological outcome according to the modified Rankin Scale (mRS) was assessed by a neurologist with special expertise in stroke in a personal visit at the stroke outpatient department or if not possible in a telephone interview at 90 days poststroke.

### Statistics

Statistical analyses were performed using IBM SPSS Statistics, version 23. The association between stroke etiology (CAS versus non-CAS) and preinterventional leptomeningeal collateral status was investigated.

Chi square test or Fisher’s exact test was used for the comparison of dichotomous variables. Parametric continuous variables were compared using the Student’s *t* test. For non-parametric data, the Mann–Whitney *U* Test was utilized.

In addition, we calculated a multivariable binary logistic regression model with favorable collaterals as the target variable. Besides CAS, it also contained age, sex and other previously identified predictors of collateral status [hypertension, M2-occlusion, baseline NIHSS and baseline Alberta Stroke Program Early CT Score (ASPECTS)] [5–7].

A *p* value less than 0.05 was considered statistically significant.

The study was approved by the ethics committee of the Medical University of Graz. Anonymized datasets generated during this study are available from the corresponding author upon reasonable request.

## Results

Over the study period, 346 patients had undergone MT for anterior circulation LVO. Of those, 65 patients were excluded due to missing CE angiography data or insufficient imaging quality (*n* = 44), ipsilateral carotid artery dissection (*n* = 9) or extracranial occlusion (*n* = 12) (Fig. 1). None of the studied patients had a significant intracranial artery stenosis.

Of the finally analyzed 281 patients (mean age 68.5 years, 48.8% female, Table 1), 46 patients had CAS-associated LVO stroke (16.4%).

**Table 1:** Clinical characteristics of anterior circulation LVO stroke patients dichotomized by stroke etiology

	MT of the anterior circulation (*n* = 281)	CAS (*n* = 46)	Non–CAS (*n* = 235)	*p* value
Demographics				
Age, years (mean, SD)	68.5 ± 12.2	67.0 ± 11.2	68.8 ± 12.4	0.342
Female [*n*. %]	137 (48.8)	15 (32.6)	122 (51.9)	0.012
Medical history (*n*. %)				
Hypertension	193 (68.7)	32 (69.6)	161 (68.5)	0.519
Dyslipidemia	51 (18.1)	9 (19.6)	42 (17.9)	0.463
Diabetes	50 (17.8)	7 (15.2)	43 (18.3)	0.398
Smoking	31 (11.0)	7 (15.2)	24 (10.2)	0.225
Atrial fibrillation	141 (50.2)	4 (8.7)	137 (58.8)	< 0.001
Previous antiplatelet therapy	73 (26.0)	18 (39.1)	55 (23.4)	0.023
Previous oral anticoagulation	40 (14.2)	3 (6.5)	37 (15.7)	0.073
Prestroke mRS (*n*. %)				0.335
0	238 (85.0)	41 (89.1)	198 (84.3)	
1	14 (5.0)	4 (8.7)	10 (4.3)	
≥ 2	28 (10.0)	1 (2.2)	27 (11.4)	
Clinical parameters				
NIHSS at presentation (median, range)	15 (4–32)	14 (7–22)	15 (4–32)	0.068
Preinterventional ASPECTS (median, min–max)	9.0 (5–10)	9.0 (6–10)	9.0 (5–10)	0.848
Symptom onset—conventional angiography (minutes; mean, SD)	199 ± 74	197 ± 85	200 ± 72	0.836
Symptom onset—reperfusion (minutes; mean, SD)	257 ± 76	264 ± 83	255 ± 75	0.555
Duration of intervention (minutes; mean, SD)	58 ± 34	67 ± 39	56 ± 33	0.081
Favorable collaterals	143 (50.9)	35 (76.1)	108 (46.0)	< 0.001
Postinterventional ASPECTS (median, min–max)	5.0 (0–10)	5.5 (0–9)	5.0 (0–10)	0.453
Infarct ≥ 2/3 MCA territory (*n*. %)	49 (17.4)	9 (19.6)	40 (17.0)	0.408
Acute stroke therapy (*n*. %)				
IV thrombolysis	166 (59.1)	25 (54.3)	141 (60.0)	0.290
MCA thrombectomy	232 (82.6)	35 (76.1)	197 (83.8)	0.146
Successful mechanical thrombectomy (TICI 2b–3)	252 (89.7)	41 (89.1)	211 (89.8)	0.533
Extracranial artery stenting^a^	11 (3.9)	11 (23.9)	0 (0.0)	< 0.001
Outcome and adverse events (*n*. %)				
NIHSS at 24 h (median, range)	10 (0–32)	12.5 (1–32)	9 (0–32)	0.291
NIHSS at stroke unit discharge (median, range)	8.5 (0–32)	6.5 (0–32)	8 (0–32)	0.141
Adverse events	24 (8.5)	9 (19.6)	15 (6.4)	0.007
Symptomatic ICH	10 (3.6)	3 (6.5)	7 (3.1)	0.231
Procedure–related artery dissection	3 (1.1)	1 (2.2)	2 (0.9)	0.416
Vessel re–occlusion	11 (3.9)	5 (10.9)	6 (2.6)	0.020
Mortality at hospital discharge	30 (10.7)	3 (6.5)	27 (11.5)	0.238
90–day mRS (median, IQR)	3 (3)	3 (3)	3 (3)	0.861
90–day mRS 0–2	134 (47.7)	21 (45.7)	113 (48.1)	0.445
90–day mRS 3–5	96 (34.2)	20 (43.5)	76 (32.3)	0.100
90–day mRS 6	51 (18.1)	5 (10.9)	46 (19.6)	0.114

*MT* mechanical thrombectomy, *CAS* Carotid artery stenosis, *SD* Standard deviation, *NIHSS* National Institutes of Health Stroke Scale, *ASPECTS* Alberta Stroke Program Early CT Score, *MCA* Middle cerebral artery, *TICI* Thrombolysis in cerebral infarction grading scale, *ICH* Intracranial hemorrhage, *mRS* Modified Rankin scale, *IQR* Interquartile range

^a^10 patients underwent carotid artery stenting simultaneously with thrombectomy; one patient was treated later on (day 38)

The remaining 235 patients (83.6%) showed a non-CAS stroke that was mainly attributed to atrial fibrillation (58.8%). Of those, no patients with ulcerated high-risk non-stenosing carotid artery plaques were observed. Compared to a non-CAS etiology, CAS-related strokes were less prevalent in female patients (32.6% vs. 51.9%, *p* = 0.012), more often pretreated with antiplatelet agents (39.1% vs. 23.4%, *p* = 0.023) and had a higher rate of favorable collaterals on pretreatment angiography (76.1% vs. 46.0%, *p* < 0.001). Patients with underlying CAS more often had postinterventional adverse events (19.6% vs. 6.4%, *p* = 0.007), while functional outcome and mortality rates were similar in both groups (Table 1). Of note, no differences were observed between CAS patients of different degree of stenosis (50–70%, *n* = 18 versus ≥ 70%, *n* = 28) in terms of collateral status, adverse events and 90-day prognosis (*p* > 0.1).

Compared to patients with unfavorable collaterals (*n* = 138, 49.1%), patients with favorable collaterals (*n* = 143, 50.9%) had a lower prevalence of AF (45.1% vs. 56.1%, *p* = 0.063), better pre- and postinterventional ASPECT Scores (pre: median, 9.0 vs. 8.5, *p* = 0.035; post: median, 6.0 vs. 4.0, p < 0.001), were more often functionally independent (mRS 0–2: 59.4% vs. 35.5%, *p* < 0.001) and were less likely deceased at 90 days (mRS 6: 7.0% vs. 29.7%, *p* < 0.001) (Table 2).

**Table 2:** Clinical characteristics of LVO stroke patients according to their preinterventional collateral status

	Favorable collaterals (*n* = 143)	Unfavorable collaterals (*n* = 138)	*p* value
Demographics			
Age, years (mean, SD)	66.6 ± 12.1	70.6 ± 12.1	0.005
Female [*n*. %]	69 (48.3)	68 (49.3)	0.864
Medical history (*n*. %)			
Hypertension	96 (67.1)	97 (70.3)	0.568
Dyslipidemia	28 (19.6)	23 (16.7)	0.526
Diabetes	22 (15.4)	28 (20.3)	0.282
Smoking	15 (10.5)	16 (11.6)	0.768
Atrial fibrillation	64 (45.1)	77 (56.1)	0.063
Prestroke mRS (*n*. %)			0.085
0	126 (88.1)	113 (81.9)	
1	10 (7.0)	4 (2.9)	
≥ 2	7 (4.9)	21 (15.2)	
Clinical parameters			
NIHSS at presentation (median, range)	14 (4–25)	16 (6–32)	< 0.001
Preinterventional ASPECTS (median, min–max)	9.0 (5–10)	8.5 (5–10)	0.035
Symptom onset—conventional angiography (minutes; mean, SD)	197 ± 86	202 ± 58	0.646
Symptom onset—reperfusion (minutes; mean, SD)	254 ± 90	260 ± 56	0.560
Duration of intervention (minutes; mean, SD)	57 ± 33	58 ± 35	0.785
Postinterventional ASPECTS (median, min–max)	6.0 (0–10)	4.0 (0–9)	< 0.001
Infarct ≥ 2/3 MCA territory (*n*. %)	13 (9.1)	36 (26.1)	< 0.001
CAS (*n*. %)	35 (31.8)	11 (11.5)	< 0.001
Acute stroke therapy (*n*. %)			
IV thrombolysis	91 (63.6)	75 (54.3)	0.113
MCA thrombectomy	115 (80.4)	117 (84.8)	0.335
Successful mechanical thrombectomy (TICI 2b-3)	131 (91.6)	121 (87.7)	0.279
Extracranial artery stenting	11 (7.7)	0 (0.0)	0.001
Outcome and Adverse events (*n*. %)			
Adverse events	10 (7.0)	14 (10.1)	0.345
Symptomatic ICH	4 (2.9)	6 (4.5)	0.481
Procedure-related artery dissection	2 (1.4)	1 (0.7)	0.583
Vessel re-occlusion	4 (2.8)	7 (5.1)	0.326
Mortality at stroke unit discharge	4 (2.8)	26 (18.8)	< 0.001
90-day mRS (median, min–max)	2 (3)	4 (4)	< 0.001
90-day mRS 0–2	85 (59.4)	49 (35.5)	< 0.001
90-day mRS 3–5	48 (33.6)	48 (34.8)	0.830
90-day mRS 6	10 (7.0)	41 (29.7)	< 0.001

*LVO* large vessel occlusion, *SD* standard deviation, NIHSS: National Institutes of Health Stroke Scale, *ASPECTS* Alberta Stroke Program Early CT Score, *MCA* Middle cerebral artery, *CAS* Carotid artery stenosis, *TICI* Thrombolysis in cerebral infarction grading scale, *ICH* Intracranial hemorrhage, *mRS* Modified Rankin scale, IQR: Interquartile range

Besides NIHSS at presentation (*p* < 0.001) and preinterventional ASPECTS (*p* = 0.033), favorable collateral status remained significantly associated with CAS-related stroke etiology in multivariable analysis (Odds ratio: 3.3, 95% confidence interval: 1.5–7.1, *p* = 0.002).

### Unfavorable collateral status and stroke etiology

138 patients had unfavorable collaterals on pre-treatment angiography (49.1%). Of those, only 11 patients had CAS-associated stroke (8.0%). Compared to non-CAS patients with an unfavorable collateral status (*n* = 127), CAS-related stroke patients with unfavorable collaterals were associated with a particularly high rate of adverse events (36.4% vs. 9.4%, *p* = 0.025) and poor outcome (median NIHSS at stroke unit discharge: 21 vs. 9, *p* = 0.037; 90-day mRS 0–2: 9.1% vs. 37.8%, *p* = 0.050).

## Discussion

This study shows that the presence of preexisting ipsilateral CAS ≥ 50% is associated with more favorable collateral status in acute LVO stroke patients. However, this does not translate into a better functional outcome at 90 days, which might be attributed to a higher rate of adverse events after MT. Although occurring rarely, CAS patients with unfavorable collaterals on pretreatment angiography face a particularly high risk of poor outcome three months after the intervention (≈ 90%).

Preinterventional leptomeningeal collaterals have a significant impact on patients’ clinical prognosis after MT. In line with earlier investigations, this study also shows that favorable collateral status on pretreatment angiography was associated with smaller postinterventional infarct size and a better functional outcome at 90 days after the intervention [5–7]. Conditions that could predict favorable leptomeningeal collaterals after acute cerebral artery occlusion are therefore of interest. In this context, chronic cerebral hypoperfusion was associated with improved cerebral collateral flow in experimental rat models and in patients with steno-occlusive disease of the carotid vasculature; and the effect increased with the degree of stenosis [4, 15, 16]. Moreover, repeated arterio-arterial (micro)embolism proceeding from aggressive carotid plaques could lead to recurrent and clinically silent cerebral ischemia, which might trigger better collaterals due to ischemic preconditioning [17].

Although recent studies only addressed primary collateral pathways in the circle of Willis, it seems plausible that CAS patients with acute intracranial LVO stroke would also be associated with favorable leptomeningeal collateral recruitment, which could further impact patients’ clinical prognosis.

While two small studies presented divergent results on that topic, [6, 7] a very recent MR-CLEAN Registry subanalysis showed that CAS-related LVO stroke patients had a better collateral status than those with cardioembolic stroke, [8] which is in line with our work. In contrast to our findings, the latter study showed slightly better median 90-days mRS scores in their CAS subgroup. This might be attributed to methodological differences between both studies: From a pathophysiological perspective, we decided to include all patients without an indication of symptomatic carotid stenosis ≥ 50% (according to the NASCET criteria) in our non-CAS group. As we did not observe patients with ulcerated carotid plaques, most non-CAS patients should have had a proximal (cardio)embolic stroke etiology, which is frequently missed on routine stroke work-up (e.g. in case of paroxysmal atrial fibrillation). However, such initially cryptogenic stroke patients are younger and have less comorbidities compared to the standard cardioembolic stroke patients [18]. The exclusion of such patients as it was done by the MR-CLEAN investigators might therefore explain the reported baseline imbalances in their subgroup analysis [8]. In contrast, our study provides comparable subgroups (CAS versus non-CAS) regarding age, medical history and prestroke mRS. Of note, the effect on outcome presented in the MR-CLEAN Registry subanalysis remained relatively low as there was no statistically significant benefit for CAS patients in terms of 90-day post-stroke dependency or mortality rates [8]. The authors concluded that larger thrombi and difficulties in gaining intracranial access due to proximal stenosis might have caused longer thrombectomy procedures compromising the positive impact of good collaterals on clinical prognosis. Our study also shows a trend towards longer interventions in CAS-related strokes, but additionally draws attention to postinterventional adverse events (predominantly vessel re-occlusions and symptomatic ICH) in CAS-related thrombectomy patients.

The high percentage of vessel re-occlusion (11%) we detected in our CAS subgroup might be also a result of more complex endovascular procedures (balloon dilatation: 43%, stenting of CAS: 24%) and residual stenosis leading to recurrent arterio-arterial embolism. Although data on vessel re-occlusion in the early phase after MT are scarce, this finding is in line with a small retrospective study, which has shown rather high rates of postinterventional re-stenosis/occlusion and a poor prognosis in LVO stroke patients with tandem pathologies [19].

Symptomatic intracerebral bleeding was the second most common adverse event in our study and might be partly attributed to extracranial artery stenting in the CAS subgroup, which required more intense antiplatelet therapy (i.e. dual antiplatelets) after the intervention. In addition, carotid artery stenting might enlarge reperfusion injury, which seems generally more pronounced in chronically ischemic tissues due to disturbances of cerebral autoregulation [9].

Of note, this is the first study that casts light on acute LVO patients with unfavorable collaterals despite underlying CAS, as they are at very high risk for postinterventional adverse events (37%) and functional dependency at 90 days (91%). Although the number of patients in this subgroup was rather small, this finding should be considered in the clinical management of such patients.

The major limitation of this study is the retrospective design and the fact that no blinding regarding stroke etiology and collateral status was possible. However, during rating, neuroradiologists were blinded to clinical information including adverse events and outcome. We decided to abstain from volumetry, but instead used pre- and postinterventional CT or MRI based ASPECT Scores to estimate the acute and final infarct, which is more feasible in daily clinical routine.

Moreover, we did not analyze the Circle of Willis for anatomical variants, which could have affected our results and should be addressed in future studies on cerebral collaterals.

Another restriction was, that not all patients underwent multi-phase CT/MR-angiography (≈ 34%). If collateral filling in the post venous phase occurs, collaterals could be underestimated when using the Tan Score in single-phase angiography. However, as this is not a frequent finding and collaterals were comparable to those of earlier investigations using multi-phase angiography, it should not have influenced our results to a major extent [20].

Finally, we cannot totally exclude that in few cases emboli broke off from the carotid stenosis leaving it less than 50%. However, patients with minor carotid artery stenosis < 50% did not meet the established ultrasound criteria for aggressive plaques (i.e. ulceration, intraplaque hemorrhage, etc.), which should exclude a major effect on the results of this investigation.

## Data Availability

Data and material from this study are available from the corresponding author upon reasonable request.
